# Co-Fermentations of Kveik with Non-Conventional Yeasts for Targeted Aroma Modulation

**DOI:** 10.3390/microorganisms10101922

**Published:** 2022-09-27

**Authors:** Kevin Dippel, Katrin Matti, Judith Muno-Bender, Florian Michling, Silvia Brezina, Heike Semmler, Doris Rauhut, Jürgen Wendland

**Affiliations:** 1 Department of Microbiology and Biochemistry, Hochschule Geisenheim University, Von-Lade Straße 1, D-65366 Geisenheim, Germany; 2 Geisenheim Yeast Breeding Center, Hochschule Geisenheim University, Von-Lade Straße 1, D-65366 Geisenheim, Germany

**Keywords:** Kveik, brewing, volatile aroma compound, maltotriose, *Saccharomyces*, *Hanseniaspora*

## Abstract

Kveik are consortia of yeast used for farmhouse ale production in Western Norway. Yeast strains derived from these mixtures are known, for example, for their high fermentation rate, thermotolerance, lack of phenolic off flavor production (POF-) and strong flocculation phenotype. In this study, we used five single cell yeast isolates from different Kveik yeasts, analyzed their fermentation and flavor production, and compared it with a typical yeast used in distilleries using 20 °C and 28 °C as the fermentation temperatures. One of the isolates, Kveik No 3, showed an impairment of maltotriose utilization and thus a reduced ethanol yield. Kveik fermentations for spirit production often harbor bacteria for flavor enrichment. We sought to improve Kveik fermentations with non-conventional yeasts (NCY). To this end we co-fermented Kveik isolates with *Hanseniaspora uvarum, Meyerozyma guilliermondii* and *Pichia kudriavzevii* using 5:1 ratios (Kveik vs. NCY) at 20 °C. The combinations of Kveik No 1 with *P. kudriavzevii* and Kveik No 1 with *Hanseniaspora uvarum* showed substantially increased amounts of specific volatile aroma compounds that were previously identified in the NCYs. Our results indicate that Kveik isolates appear to be suitable for co-fermentations with certain NCY to enhance beer or spirit fermentations, increasing the potential of these yeasts for beverage productions.

## 1. Introduction

Yeasts of the genus *Saccharomyces* have a long history of domestication due to their constant use in baking and alcoholic beverage production [1]. Accordingly, *S. cerevisiae* may be considered mankind’s first domesticated species [2]. Domestication, as seen in lager yeast, worked efficiently since a suitable niche was provided ensuring constant nutrient supply and storage [3]. Lager yeast strains evolved as hybrids from *S. cerevisiae* and *S. eubayanus,* and today two main groups are recognized as Saaz and Frohberg, which evolved from a single hybridization event [3,4]. These groups can be distinguished based on their ploidy with Saaz yeasts being~triploid and Frohberg yeast~tetraploid with added aneuploidies for individual chromosomes [5,6]. Lager beer fermentations require yeast strains that ferment at low temperatures, utilize maltose and maltotriose (for which Saaz yeasts have a higher consumption rate), produce clean aromas, i.e., no phenolic off-flavors, and flocculate well, i.e., collect at the bottom of the brewing vessel at the end of fermentation [7].

Ale yeasts, on the other hand, are mainly *S. cerevisiae* yeasts that cannot utilize melibiose in contrast to lager yeasts and ferment at higher temperatures (15 °C–25 °C) due to higher maltose transport activity at these temperatures [8].

A group of farmhouse ale yeasts, used for centuries in an isolated region of Western Norway, are known as Kveik [9]. Kveik represent yeasts or consortia of different yeast strains (including bacteria) that were selected for their fast fermentation and short lag phase (hence ‘Kveik’ as in ‘quick’), high temperature and high ethanol tolerance, maltose utilization, high gravity fermentation, POF- negative phenotype and the ability to withstand drying on Kveik rings until the next fermentation cycle, potentially occurring a few months later. Traditionally, Kveik was pitched into warm wort of >30 °C, which is drastically different from other beer brewing conditions [9,10].

A comparative genome analysis of six Kveiks indicated that they are tetraploid *S. cerevisiae* yeasts that belong to the ‘Beer 1’ clade composed of yeasts from Germany, the UK and the US. However, they also contain a *S. cerevisiae* hybridization partner that is yet unknown [9]. Several fermentation traits could be related to their genomic make-up: fast maltose utilization based on amplification of *MAL* genes, POF- phenotype based on loss-of-function mutations in either *PAD1* or *FDC1* required for the decarboxylation of hydroxycinnamic acids to 4-vinylguaiacol, and a high flocculation rate based on copy number variations in the flocculation genes *FLO1* and *FLO11* [9]. The accumulation of intracellular trehalose may contribute to the high fermentation temperature tolerance and high ethanol tolerance of Kveiks [10]. One isolate from a Kveik culture, Muri, was revealed to be an allodiploid hybrid of *S. cerevisiae* and *S. uvarum,* indicating that some degree of diversity can be expected [11].

As Kveiks adapt to high temperature fermentations, they have a strong potential to be used in a variety of fermentations that then are sufficient without cooling. Besides energy cost saving, faster fermentation speed of Kveiks may also free up capacity in breweries. Kveiks (Hornindal, Lida) have been used in stout beer and Indian Pale Ale fermentations and have generated more fruity aromas than a commercial control strain [12,13].

Non-sterile fermentations, such as in whisky production, require fast fermenting strains with high temperature and high ethanol tolerance. The presence of bacterial strains is tolerated and may be associated with positive aroma contribution [14,15]. The limitations of the use of *Saccharomyces* in fermentations, compared to some successful spontaneous fermentations that are based on a much broader biodiversity, have led to an interest in employing NCYs in beverage productions. Studies on NCYs have been increasing over the past two decades with a focus on lowering the ethanol content and/or enrichment of flavors in the final beverage [16,17,18,19,20,21]. Recent publications have highlighted, for example, the use of *Wickerhamomyces anomalus* in co-fermentations with *S. cerevisiae* strains to enhance the flavors in beer and wine [22,23].

Previously, we isolated and characterized a series of NCYs and identified *H. uvarum, M. guilliermondii* and *P. kudriavzevii* as potentially beneficial strains for co-fermentations [24]. In this study, we examined if beer yeast flavor can be specifically modulated by these NCYs, enriching the fermented beverages with fruity esters. To this end, we used five Kveiks obtained from a whisky distillery, obtained single colonies thereof and characterized their fermentation properties at 20 °C and 28 °C. Thereafter, we employed these Kveiks and the three above-mentioned NCYs in co-fermentations of unhopped granmalt and analyzed their fermentation progress, sugar utilization and aroma compound formation. This revealed specific Kveik—NCY combinations that may be validated in up-scaled fermentations.

## 2. Materials and Methods

### 2.1. Strains and Media

Yeast strains were subcultured in YPD (1% yeast extract, 2% peptone, 2% glucose). The YPD plates for single colony isolation using the streak plate method contained 2% agar. Unhopped and rehydrated granmalt (GranMalt AG, Bergkirchen, Germany) was prepared at 15° Plato and sterilized by autoclaving. The same batch was used for all experiments. The sugar content of the malt was determined by HPLC (High-Performance Liquid Chromatography) and is shown in Table 1 (carried out as described in [24]).

For sporulation, assays cells were precultured in liquid YPD, centrifuged and washed with sterile water and then streaked out on solid sporulation media (2% potassium acetate, 2% agar; pH 6.5–7.0). Plates were incubated for 10 days at 25 °C before determining the sporulation efficiency. The strains used in this study are outlined in Table 2.

### 2.2. Microscopy and Micromanipulation

Microscopic images of Kveiks and ale yeasts were generated using Differential Interference Contrast (DIC) on a Zeiss AxioImager M2 (version number, Jena, Germany) equipped with a Plan-Apo 100x/1.4 Oil objective. Images were acquired with a pce.edge 4.2 bi digital camera and processed using Fiji software (version number, City, Country) [25].

Micromanipulation of yeast tetrads was carried out on an MSM System 400 (Singer Instruments, Roadwater, UK). A loopful of cells from a sporulation plate were resuspended in 45 µL sterile water and treated with 5 µL zymolyase (10 mg/mL) for 30 min at 30 °C. A portion of 10 µL was streaked out on the inoculation field for tetrad dissection.

### 2.3. Fermentation Conditions

Lab scale fermentations were carried out in 250 mL tall-tube cylinders filled with 150 mL Granmalt wort. Wort was pitched with yeasts at 1 × 10^7^ cells/mL. Fermentation temperatures were kept constant at either 20 °C or 28 °C. Fermentations were stirred constantly at 300 rpm. The fermentation progress was monitored by documenting mass loss due to CO_2_ release. Co-fermentations of Kveiks with NCYs were carried out at 20 °C. The fermentations were carried out with initial ratios of one part NCY and five parts Kveik. Appropriate dilutions were generated based on OD_600 nm_ measurements. All fermentations were carried out in triplicate.

### 2.4. Analytical Methods

The end of fermentation was reached when mass loss and CO_2_ release reached a plateau. Sugar consumption and residual sugar concentrations were measured by HPLC at the end of fermentation as described [24,26]. Volatile aroma compounds (VACs) were determined by headspace solid phase micro extraction and gas chromatography coupled with mass spectrometry (HS-SPME-GC-MS) based on methods described previously [26,27].

### 2.5. Statistical Analyses

To obtain estimates of relative effect sizes of (A) the Kveik strain and (B) the NCY strains on volatile aroma composition, we performed redundancy analysis (RDA) on the VAC data set (see Supplemental Material). Statistical significance of the VAC increase in co-fermentations was determined by the Wilcoxon rank sum test (one-sided tests, comparing VAC amounts from co-fermentations to corresponding values from a Kveik single fermentation) for each VAC. Statistical analyses were performed in R version 4.0.3 (R Core Team 2020, Vienna, Austria).

## 3. Results

### 3.1. Kveiks Differ from Ale Yeasts in Their Germination Efficiency of Spores

**Figure 1:**
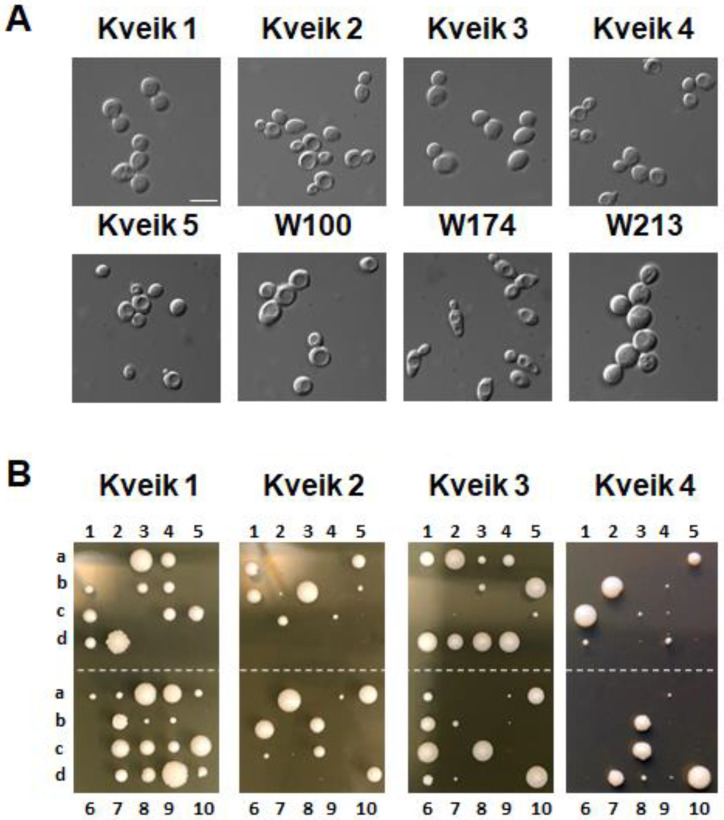
Life cycle assessment of Kveiks. (**A**) The indicated strains were grown in liquid YPD and imaged using differential interference contrast optics. Size bar is 10 µm. (**B**) Cells of the same strains as in (**A**) were sporulated on solid media and ten tetrads (two rows of five tetrads each) of each strain were dissected by micromanipulation on YPD plates. Plates were incubated for 5 days at 30 °C prior to photography.

Kveiks are described as farmhouse ale yeasts. To compare their life cycles with ale yeast production strains we used five Kveiks (see Table 2); we generated single colonies thereof and cultured them in liquid full medium (Figure 1A). These cultures were induced to sporulate on solid media and sporulation efficiency as well as germination efficiency were determined (Figure 1B, Table 3).

The ale yeasts that were studied showed quite a variable degree of sporulation, but sporulation can reach a very high level, as seen in W174 (and other ale yeasts; our unpublished results). Nevertheless, the germination efficiency of ale yeast spores was surprisingly low, making ale yeasts generally difficult breeding stocks. Kveiks, on the other hand, sporulated quite well and the germination efficiency was considerably higher, as has also been observed by others [9]. Kveik 5, however, was more similar to regular ale yeasts with a low spore germination frequency. Tetrad dissection indicated differential germination or growth of Kveik spores of individual asci. For breeding purposes, it will be interesting to analyze the fast-growing Kveik spore clones and assess their mating ability. Our comparison demonstrated that Kveiks represent unique breeding stocks among ale yeasts, which may be useful for strain improvement strategies for a variety of yeast strains.

### 3.2. Fermentations with Kveiks Are Faster at Higher Temperature and Show Differential Maltotriose Utilization

**Figure 2:**
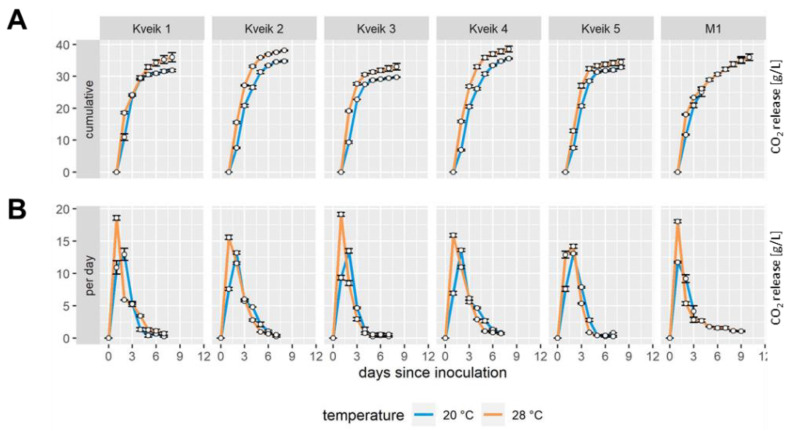
Fermentation characteristics of Kveiks. (**A**). Fermentation curves of the indicated strains and M1 control based on CO_2_-release and concomitant mass loss, which was measured daily. (**B**). Amount of CO_2_-release per day illustrates the fermentation intensity of the strains. White circles mark the mean value of three replicate fermentations; error bars are the standard error of the mean.

Kveiks are able to ferment rapidly at high temperature. However, in other brewing settings lower temperatures are employed. Thus, we studied the fermentation progress and the sugar utilization of Kveiks at 20 °C and 28 °C. Kveiks showed strong fermentation intensities at the start of fermentation and finished fermentation a day earlier at 28 °C, compared to the 20 °C fermentations (Figure 2A). Fermentation intensity was very high in the first few days and then rapidly declined. The Kveiks’ fermentation behavior was comparable to the M1 distillery strain, which also showed a rapid fermentation start but required longer to reach the fermentation end point (Figure 2B).

A complete utilization of available sugars is required for maximal ethanol yield. We quantified wort sugars (glucose, fructose, and sucrose as well as maltose and maltotriose) using HPLC. This indicated that Kveiks, particularly Kveik 3, did not fully consume maltotriose. While Kveik 3 consumed only 25–30% of maltotriose at the tested temperatures, the other Kveiks utilized between 81% and 91% of the maltotriose. The M1 strain outperformed the Kveiks in terms of maltotriose utilization. However, M1 could not consume all of the maltose at a higher temperature (only 88%), in contrast to the Kveiks. As maltose is the major wort sugar, overall sugar consumption was higher in the Kveiks, except for Kveik 3. The amount of ethanol produced by Kveiks was in the expected range, yet, was found to be lower than that of M1. Overall, the ethanol yield was higher at 20 °C than at 28 °C (Table 4).

### 3.3. Volatile Compound Formation of Kveiks at Different Fermentation Temperatures

Our fermentations are routinely analyzed for the major esters and higher alcohol volatile aroma compounds. Fermentations of Kveiks at 20 °C were compared with M1 fermentations. The most abundant ester by far in these fermentations was ethyl acetate (acetic acid ethylester) in the range of 37 to 95 mg/L. M1 produced almost twice as much ethyl acetate as the Kveiks. The other major esters were isoamyl acetate (acetic acid 3-methylbutylester), ethyl octanoate (octanoic acid ethylester) and 2-phenyl acetate (acetic acid phenylethylester), which made up 67–84% of all other esters in these strains.

Kveik 3 and M1 were similar in their isoamyl acetate production, but overall, the Kveiks generated substantially less esters compared to M1, with Kveik 1, 2 and 4 reaching around 60% and Kveik 5 about 75% of the levels found in M1. Kveik 3 was different in that it produced a high amount of isoamyl acetate and 2-phenyl acetate, which are both products derived from Ehrlich pathway degradation of amino acids leucine and phenylalanine, respectively (see Table S1).

Although M1 outperformed the Kveiks in the production of esters, the profiles for higher alcohols (e.g., isoamyl alcohol and 2-phenyl ethanol) were very similar in all strains and amounted to a total of 250–320 mg/L, but still with M1 at the higher end. The major acids detected were i-valeric acid and decanoic acid (Table S1).

In fermentations at 28 °C, M1 again produced the highest amount of ethyl acetate, ~twice as much as Kveiks 1, 4, 5 and ~50% more than Kveiks 2 and 3. However, the amount of other aroma esters generated by M1 was considerably lower at 28 °C compared to the 20 °C fermentations (only 40%), suggesting lower production or retention at higher fermentation temperatures. The Kveiks performed quite heterogenously. Kveik 1 was similar to M1 in that it showed an overall reduction of ester compounds at 28 °C, compared to 50% of that at 20 °C fermentations. Kveik 2, on the other hand, performed at 28 °C: very similar to the 20 °C fermentations. The only major change was a reduction in isoamyl acetate production in this strain, which was contrasted by an increased ethyl decanoate (decanoic acid ethylester) production. Kveiks 3, 4 and 5 showed smaller reductions in ester compounds: 76%, 83% and 95% of the 20 °C levels, respectively, also with substantial reductions in isoamyl acetate production (Table S2).

The production of higher alcohols at 28 °C was very similar to the production at 20 °C. Interestingly, under these conditions M1 was found at the lower end of the scale (240 mg/L). The major higher alcohol at both fermentation temperatures was isoamyl alcohol, accounting for ~50% of the higher alcohols. The amounts of isovaleric acid were similar under both fermentation conditions (0.9–1.7 mg/L; Table S2). This is within the range found in ale yeasts also used in this study, while lager yeasts are at the lower end of this range (not shown).

### 3.4. Co-Fermentations of Kveiks with Selected Non-Conventional Yeasts

**Figure 3:**
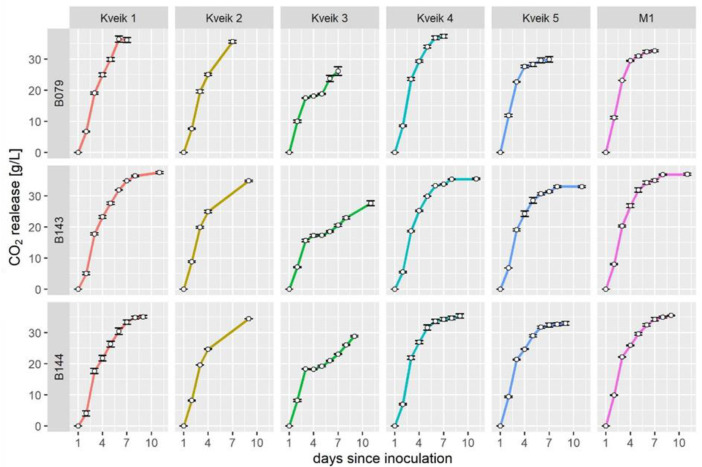
Co-fermentation of Kveiks with NCYs using granmalt wort at 20 °C. The five Kveiks were co-fermented with *H. uvarum* (B079, top row)), *P. kudriavzevii* (B143, middle row) and *M. guilliermondii* (B144, bottom row) at a ratio of 5:1. Mass losses were determined daily by weighing the tall tube fermenters. White circles mark the mean value of three replicate fermentations; error bars are the standard error of the mean.

At higher temperatures the Kveiks (except for Kveik 1) generated substantially higher amounts of esters (except ethyl acetate) than M1. However, overall production of esters was improved at lower temperatures in all strains and M1 outperformed the Kveiks at 20 °C. Therefore, we aimed for a targeted improvement of Kveik flavor output by adding NCYs in co-fermentations. Previously, we characterized three NCYs, *Hanseniaspora uvarum, Meyerozyma guilliermondii* and *Pichia kudriavzevii*, in grape must fermentations [24]. These yeasts showed good alcohol tolerance and fast fermentation in high sugar must. The major aroma esters found in these NCYs were ethyl propionate (propanoic acid ethylester) and ethyl (i-)butyrate ((i-)butyric acid ethylester), which impart pineapple-like odors [28]. As these esters were not prominent in Kveik aroma profiles, we hypothesized that co-fermentations of the Kveiks with each of the NCYs could specifically alter and improve their aroma profiles. Co-fermentations were carried out in granmalt wort, as before, and at 20 °C, a temperature which is equally well suited to the NCY strains. We did not aim for an overly pronounced impact of the NCYs, particularly because of their marked potential to generate ethyl acetate. Therefore, we used a ratio of 5:1 in favor of the Kveiks in these co-fermentations. Each of the five Kveiks was fermented with each of the three NCYs in triplicate samples (Figure 3).

The co-fermentations showed a vigorous start and slowed down after three days to reach the end of fermentation after ~9 days. In all co-fermentations Kveik 3 could not perform well after day 3 and fermentations halted for the three following days to then gradually pick up again (Figure 3). As shown in single fermentations, Kveik 3 was impaired in maltotriose utilization, which could not be compensated by any of the NCYs. In the co-fermentations Kveiks regularly used up >90% of the maltotriose and consumed all of the maltose (except for Kveik 4 in the *H. uvarum* co-fermentation where a residual 3% of the maltose was found at the end of the experiment; see Table S3). In all Kveik 5 co-fermentations less CO_2_ was released compared to Kveiks 1,2 and 4, which was also reflected in their lower ethanol yields (Table 5).

The M1 strain performed well in all co-fermentations. With M1-*H. uvarum* co-fermentations the total ethanol production was slightly reduced compared to M1-solo fermentations, while co-fermentations with *P. kudriavzevii* slightly enhanced ethanol production. The Kveiks 1, 2 and 4 produced slightly lower ethanol yields in co-fermentations with B143 and B144 than with M1, but performed better than M1 in co-fermentations with B079/*H. uvarum* (Kveik 1 and 4 with 10% increased ethanol yields).

**Figure 4:**
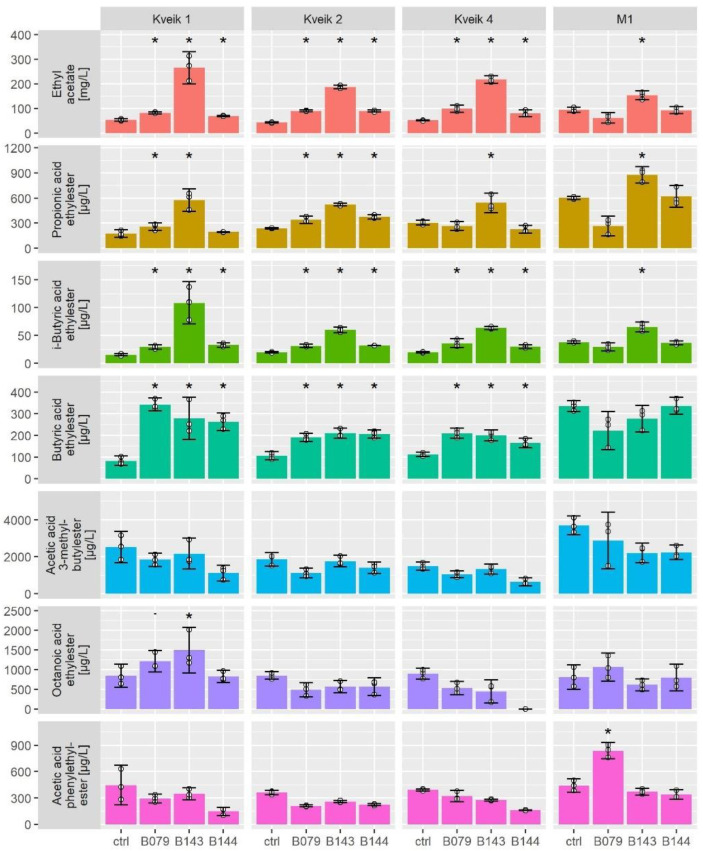
Amounts of selected volatile aroma compounds of Kveiks and M1 single and co-fermentations. Bar height is the mean of 3 replicate fermentations. Error bars show 80% quantile interval around the mean; white points are the measured values. Asterisk: difference to control was significant ≤0.05; dot: difference to control was significant ≤0.10. B079—*H. uvarum*; B143—*P. kudriavzevii*, B144—*M. guilliermondii*; ctrl—single fermentation of Kveik or M1.

As Kveik 3 and Kveik 5 did not perform satisfactorily, we focused on a comparison of aroma profiles of co-fermentations of Kveiks 1, 2 and 4 with those of M1. In these co-fermentations we aimed for an increase in propanoic acid ethylester, butyric acid ethylester and i-butyric acid ethylester via the NCYs, which actually could be demonstrated (Figure 4).

The M1-B143 co-fermentations revealed that flavor in this distillery strain could also be modulated in this targeted way. The co-fermentations led to an increase in ethyl acetate production, e.g., in the Kveik 1—*P. kudriavzevii* example up to five-fold. However, the ethyl acetate production remained at a much lower level than seen before in NCYs’ single fermentations [24]. Co-fermentations had a negative effect on the number of higher alcohols that were generated by Kveiks. The M1 strain, however, was far less affected, particularly the co-fermentations with *H. uvarum* and the *P. kudriavzevii* generated higher amounts of 2-phenyl ethanol.

The Kveik 1—*P. kudriavzevii* co-fermentations produced a substantial increase in volatile aroma compounds over the Kveik 1 single fermentation, which can mostly be attributed to the input of the desired NCYs esters. However, the generation of flavor compounds based on amino acid metabolism through the Ehrlich pathway was negatively affected in most co-fermentations [29]. This comprises the higher alcohols isoamyl alcohol (3-methyl-butanol) and 2-phenyl-ethanol and their esters acetic acid 3-methylbutylester and acetic acid phenylethylester. The co-fermentation of Kveik 1 with *P. kudriavzevii* showed the overall best results with increased ethanol and aroma ester production, outcompeting all other strains, including M1, at 20 °C. The runner-up in these trials was the Kveik 1 co-fermentation with *H. uvarum* (Figure 4 and Table S3).

## 4. Discussion

Interest in Kveiks has surged only recently due to their favorable fermentation traits. These yeasts evolved in particular niches as farmhouse ale yeasts in Western Norway for centuries. Survival in these environments required specific domestication events. As a result, Kveiks initiate fermentation of high gravity wort very fast (‘quickly’), ferment well at high temperatures(>30 °C), tolerate high ethanol levels, produce no phenolic off-flavors, flocculate abundantly and tolerate drying and dry storage [9]. Kveiks are tetraploid as a result of hybridization of a parent from the ‘beer 1’ clade with an unknown other yeast parent [10]. Interestingly, Kveiks tested in this study retained a good sporulation and spore germination efficiency. This may be due to their infrequent use and dry storage, which may have been selected for sexual reproduction and sporulation to survive these periods. The Kveiks used in our study excelled ale yeasts in spore viability/spore germination efficiency.

Ale yeasts may sporulate abundantly but show a rather low spore viability [30]. The poor spore germination rate of ale yeasts impedes their use in breeding programs. Therefore, Kveiks with their beneficial fermentation properties will make interesting breeding stocks in the future. The genetic basis for some of the Kveik fermentation traits has been analyzed using comparative genomics, while the increased thermo- and ethanol tolerance of Kveiks has been linked with the ability to accumulate trehalose intracellularly [9,10].

Kveiks will now certainly be tested in all different brewing and baking environments, and first reports on Kveiks used for Indian Pale Ale and Stout beer and wine fermentation have been published [12,13,23]. Our study is based on Kveiks derived from a distillery environment. In whisky production yeast strains with optimal maltose and maltotriose utilization are required. Kveiks were shown to have difficulties in completely consuming maltotriose, and transport of maltose has been shown to be temperature dependent [8,10]. In our study Kveik 3 was severely impaired in maltotriose utilization at 20 °C and 28 °C. Strain M1 consumed all maltose and maltotriose under these conditions while the other Kveiks did not always finish maltose and used only 80–90% of the maltotriose. However, this malus of the Kveiks can easily be remedied with conventional yeast breeding, for example, with an ale yeast mating partner as group I lager yeasts often are also impaired in using maltotriose [31].

Aroma compounds may enter whisky upon distillation. Therefore, yeasts that generate copious volatile aromas may be favorable. Flavor contributions of other organisms have been viewed as spoilage, but recently they have also been looked at more positively [15,32]. The use of different lactic acid bacteria (LAB) in malt whisky fermentations has been studied. These bacteria may develop as the wort is not boiled in contrast to beer fermentations and selection of *Lactobacilli* may contribute beneficial flavors to whisky [14,33]. Particularly, ethyl butanoate has been valued for its positive influence on the whisky aroma with its fruity flavor (orange, pineapple) [15,34]. However, inclusion of LAB will result in the formation of lactic acid and its ester ethyl lactate (buttery, creamy odor), which did not occur in our co-fermentations with NCYs. In our Kveik—NCYs co-fermentation a decrease in Ehrlich pathway degradation products was observed. The Ehrlich pathway describes the degradation of amino acids, e.g., leucine and phenylalanine [29]. This suggests that in Kveik—NCY interactions there is a competition for the available amino nitrogen. This may come as a surprise as all malt wort was thought to contain a sufficient N-supply. Transferals of these Kveik—NCY co-fermentations to other systems, e.g., grape must, should therefore consider supplementation of yeast available nitrogen beyond the regular 150 mg/L total nitrogen.

NCYs have been employed in different co-fermentation regimes with *S. cerevisiae*, e.g., for lowering final ethanol content, for contributing aroma-related compounds or antioxidant activity, for producing craft beers or low-calorie beers, and to enhance glycerol production [18,35,36,37,38,39,40]. Different yeast genera repeatedly were highlighted, e.g., *Brettanomyces, Hanseniaspora, Kluyveromyces, Pichia, Meyerozyma, Torulaspora* or *Zygosaccharomyces* [41,42]. *H. uvarum* was tested in co-fermentations of grape must with *S. cerevisiae*, while *P. kudriavzevii* was shown to have some potential to co-ferment an overripe Cabernet Sauvignon grape must [43,44]. As with spontaneous fermentations, co-fermentations often are unpredictable in their outcome, thus impeding targeted flavor adaptation.

In our co-fermentations, nearly all observed variation in VAC-production could be explained as a result of these co-fermentations in redundancy analyses. Since every combination of Kveik × NCY constituted a distinct flavor profile, we focused on separate analysis of the overall effects of Kveik strains and NCY strains; both variables were significant predictors of VAC composition. The overall impact on the aroma output in these co-fermentations was larger for the NCY strains (60% contribution) and less for the Kveik strains (25%; *p* = 0.01), respectively (Table S4, Figures S1 and S2).

There are few successful examples of targeted impact on flavor formation: (i) use of high-gravity wort to increase acetate ester production [45]; (ii) the addition of leucine to wort to increase the formation of isoamyl acetate [46]; or (iii) the use of mutants selected for resistance against the toxic amino acid analog tri-fluoro leucine, which also results in increased isoamyl alcohol production [47,48].

## 5. Conclusions

We have characterized Kveiks in wort fermentations and we were able to improve ester compound formation by the co-fermentation of Kveiks with NCYs. This represents a successful example of targeted flavor manipulation in a fermented beverage using specific pre-selected NCYs. The NCYs impacted the flavor output independently of the Kveiks tested. It is thus likely, that these NCYs will be suitable also in a wider range of *Saccharomyces* co-fermentations. In particular, our study opens the way for larger scale trials using the specific strain pairs Kveik 1/*H. uvarum* and Kveik 1/*P. kudriavzevii* in co-fermentations.

## Figures and Tables

**Table 1:** Sugar content of rehydrated granmalt used in this study.

	Glucose [g/L]	Fructose [g/L]	Maltose [g/L]	Sucrose [g/L]	Maltotriose [g/L]	Total Sugars [g/L]
Wort	18.7	11.8	43.7	29.1	14.2	117.5

**Table 2:** Strains used in this study.

GYBC *	Strain +	Description	Source
145	Kveik No 1	*Framgarden*	Aurora Spirit Distillery, Lyngseidet, Norway
146	Kveik No 2	*Strada*	
147	Kveik No 3	*Tormodgarden*	
148	Kveik No 4	*Ebbegarden*	
149	Kveik No 5	*Hornindal*	
	SafSpirit M1	Distillery yeast	Fermentis, Marcq-en-Barœul, France
214	*Hanseniaspora uvarum B079*		[24]
215	*Meyerozyma guilliermondii B143*		[24]
216	*Pichia kudriavzevii B144*		[24]
	W100	Ale yeast	Hefebank Weihenstephan GmbH, Hallertau, Germany
	W174	Ale yeast	
	W213	Ale yeast	

* GYBC: Geisenheim Yeast Breeding Center; +: The Kveik strains 1–5 are single cell isolates of the original Kveiks provided by the Aurora Spirit Distillery.

**Table 3:** Germination efficiency of Kveik and ale yeast spores.

Strain	Sporulation *	Germination +
Kveik 1	78%	64%
Kveik 2	68%	66%
Kveik 3	31%	62%
Kveik 4	57%	36%
Kveik 5	43%	9%
W100	20%	8%
W174	90%	4%
W213	30%	3%

* only four-spored asci were scored. Cells were kept on sporulation plates for 10 days and 400 cells were analyzed. + For determining the germination efficiency 100 spores derived from 25 tetrads were dissected by micromanipulation (see Figure 1B).

**Table 4:** Residual sugars and total ethanol content.

Strain	Glucose [g/L]	Fructose [g/L]	Maltose [g/L]	Sucrose [g/L]	Maltotriose [g/L]	Total Sugars [g/L]	Ethanol [g/L]	Ethanol [%]
Fermentations at 20 °C								
**Kveik 1**	<1	<1	<1	<1	2.0 ± 0.7	2.0 ± 0.7	50.7 ± 1.1	6.4 ± 0.1
**Kveik 2**	<1	<1	<1	<1	1.3	1.3	56.4 ± 0.9	7.1 ± 0.1
**Kveik 3**	<1	<1	<1	<1	10.0 ± 0.2	10 ± 0.2	47.3 ± 0.3	6.0
**Kveik 4**	<1	<1	1.0 ± 0.1	<1	1.2	1.9 ± 0.6	55.4 ± 0.3	7.0
**Kveik 5**	<1	<1	2.7	<1	2.7 ± 0.2	3.5 ± 1.7	50.7 ± 0.4	6.4
**M1**	<1	<1	<1	<1	<1	<1	58.5 ± 1.8	7.4 ± 0.2
**Fermentations at 28 °C**								
**Kveik 1**	<1	<1	<1	<1	2.6 ± 0.8	2.6 ± 0.8	42.4 ± 1.4	5.4 ± 0.2
**Kveik 2**	<1	<1	1.0 ± 0.1	<1	1.2	2.2 ± 0.1	47.1 ± 0.4	6.0 ± 0.1
**Kveik 3**	<1	<1	<1	<1	10.6 ± 0.6	10.6 ± 0.6	39.5 ± 0.9	5.0 ± 0.1
**Kveik 4**	<1	<1	<1	<1	1.2 ± 0.1	1.2 ± 0.1	46.4 ± 0.9	5.9 ± 0.1
**Kveik 5**	<1	<1	<1	<1	2.1 ± 0.4	2.1 ± 0.4	44.1 ± 0.1	5.6
**M1**	<1	<1	5.3 ± 0.9	<1	<1	6.3 ± 0.9	53.4 ± 1.8	6.8 ± 0.2

**Table 5:** Ethanol content (%) the end of Kveik + NCY co-fermentations.

	Single	B079	B143	B144
K1	6.4 ± 0.1	7.7 ± 0.3 *	7.7 ± 0.1 *	7.0 ± 0.1 *
K2	7.1 ± 0.1	7.3 ± 0.1 +	7.3 ± 0.1 +	7.1 ± 0.2
K3	6.0 ± 0.0	5.5 ± 0.8	6.0 ± 0.4	5.7 ± 0.1
K4	7.0 ± 0.1	7.8 ± 0.1 *	7.4 ± 0.3 +	7.0 ± 0.1
K5	6.4 ± 0.1	6.3 ± 0.4	6.9 ± 0.1 *	6.5 ± 0.1 +
M1	7.4 ± 0.2	7.0 ± 0.0	7.7 ± 0.1	7.4 ± 0.1

* and +: ethanol levels were increased compared to Kveik single fermentations with *p* < 0.05 and *p* < 0.10, respectively (Wilcoxon test).

## Data Availability

Not applicable.

## Supplementary Materials

The following supporting information can be downloaded at: https://www.mdpi.com/article/10.3390/microorganisms10101922/s1, Table S1: Aroma data of 20 °C-fermentations; Table S2: Aroma data of 28 °C fermentations; Table S3: Aroma data of co-fermentations; Table S4, Figures S1 and S2: additional statistical analyses. References [49,50,51] are cited in the supplementary materials.
