# Supplementary material for: Co-Fermentations of Kveik with Non-Conventional Yeasts for Targeted Aroma Modulation
Source: Microorganisms. 2022 Sep 27;10(10):1922. doi: 10.3390/microorganisms10101922 (PMC9612206; doi:10.3390/microorganisms10101922)
Supplement: Supplementary file 1 [file microorganisms-10-01922-s001.zip › microorganisms-1899060-supplementary/Table S4 - Figures S1+S2.pdf]

Supplemental Material

Redundancy Analysis

Volatile aroma compound (VAC) data were Hellinger transformed (Legendre and Legendre, 2012, p. 327-333; Legendre and Gallagher, 2001). The data comprised 26 analytes. Homogeneity of multivariate variances (HMV) of the transformed data were tested with functions betadisper and permutest from R package vegan (Oksanen et al. 2020). HMV was not rejected for factors Kveik yeast strain (levels K1, K2, K4 and M1) and NCY strain (levels B079, B143 and B144) (Table S4a). We thus proceeded with redundancy analysis (RDA, Legendre and Legendre 2012, p. 656-661). In all instances p-values were obtained by 99 random permutations of the VAC data.

Results

We focussed our analysis on the overall effects of Kveik strains and NCY strains (Table S4a). Figures S1 and S2 show co-fermentations in RDA space. For visual clarity, NCY strains and Kveik strains were highlighted separately (Figures S1 and S2 respectively). Both NCY strain and Kveik strain were statistically significant predictors of flavour composition (Table S4b). NCY strains had a larger impact on flavour composition than Kveik strains (60 % and 25 % respectively, Table S4b). Approx. 15 % residual variation remained unexplained by this simple model. The latter comprised VAC quantification error and random microbiological variation during fermentations.

We did not consider interactions between Kveik strain and NCY strain. When analysed, they explained > 95% of total variation. In other words: Variation in VAC composition could be explained almost entirely by any particular combination of Kveik strain and NCY strain. It follows, that (almost) any combination of Kveik and NCY strains constituted a distinct flavour profile (*cf.* panel A Figure S1). Here, residual variance (error variance or unexplained variance) was very small (< 5%).

Table S4a. Results Homogeneity of multivariate variances volatile aroma compound data

Explanatory Variable	Model Sum of Squares	Model Mean Squares	Residual Sum of Squares	Residual Mean Squares	F statistic (within group df, residual df)	p(>F)*
Kveik strain	0.009451	0.0031505	0.195193	0.0060998	0.5165 (3, 3)	0.76
NCY strain	0.001038	0.00051884	0.064629	0.00195845	0.2649 (2, 33)	0.87

*: df – degrees of freedom; p (>F) – probability that, while H0 (equal variance among groups) is true, the value of the F statistic is larger than observed

Table S4b. Results of redundancy analysis of volatile aroma compound data

Explanatory Variable	Explained variance	% of total Variation	F statistic (within group df, residual df)	p(>F)
Kveik strain	0.0041672	25.01111	16.647 (3, 3)	0.01
NCY strain	0.0099905	59.96	59.866 (2, 33)	0.01
residual	0.0025032	15.02		
total	0.0166609			

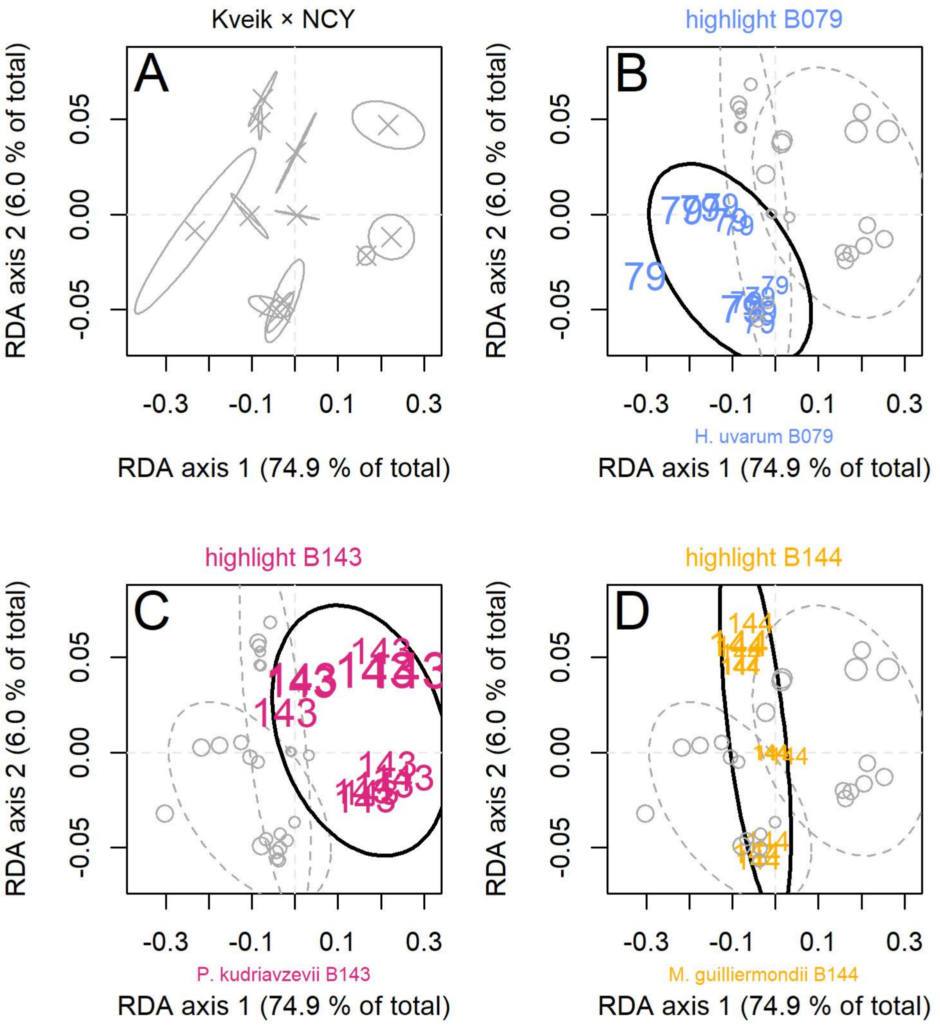

Figure S1. Ordination (redundancy analysis, RDA) of Kveik co-fermentations based on Hellinger transformed volatile aroma compound composition data (26 analytes). All panels show the same ordination (i.e. scatter of points) highlighting different sets of co-fermentations; here: NCY strains. **(A)** general overview: crosses mark centroid of replicate co-fermentations (N=3) for each unique combination of Kveik and NCY strain; ellipses are 80 % confidence ellipses based on standard deviation. **(B,C,D)** highlight NCY strains: Notice pronounced clustering of the latter along the first RDA axis.

Non-highlighted co-fermentations are shown as grey circles; non-highlight confidence ellipses with hashed lines; size of symbols and characters is proportionate to total volatile aroma content of a co-fermentation. Percentages of total variation (RDA axes labels) is the proportion of total variance observed in the transformed VAC data which correlated with independent variables NCY strain and Kveik strain in RDA. NCY strains: B079 – *Hanseniaspora uvarum* B079 (blue); B143 – *Pichia kudriavzevii* B143 (magenta), B144 – *Meyerozyma guilliermondii* B144 (orange).

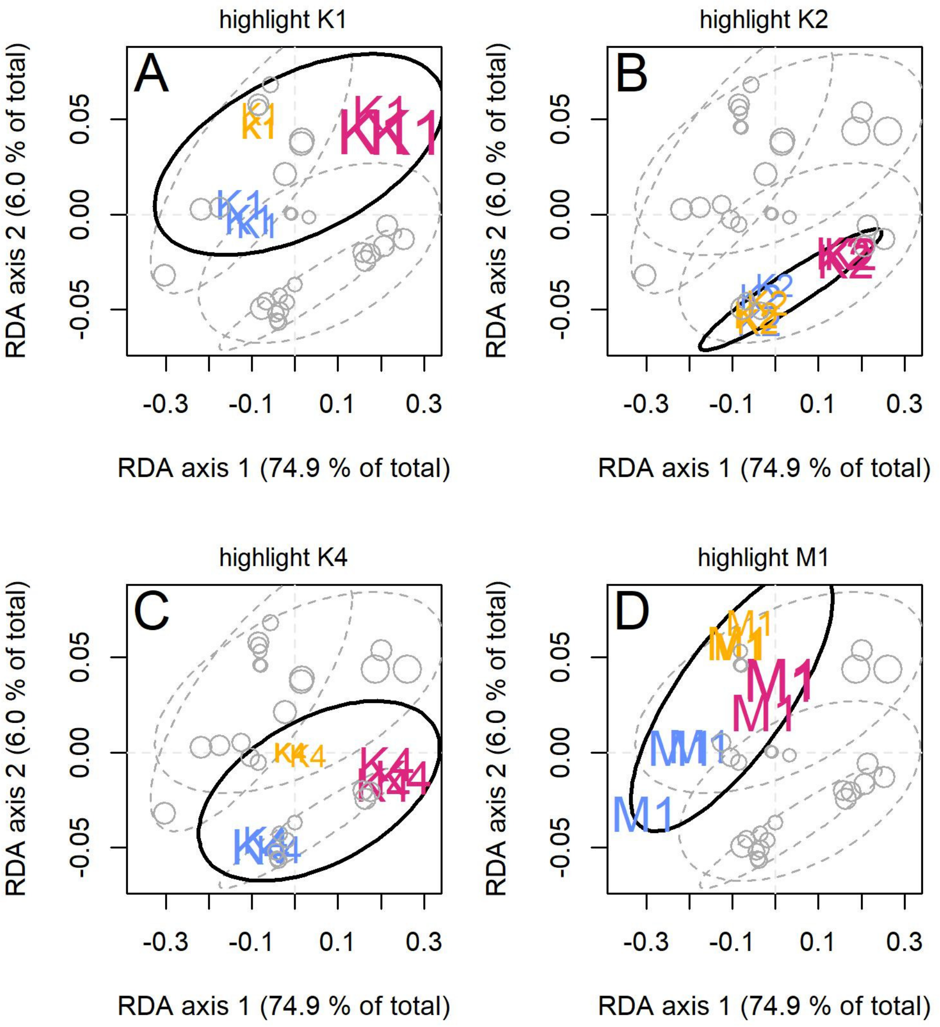

Figure S2. Ordination (redundancy analysis, RDA) of Kveik co-fermentations based on Hellinger transformed volatile aroma compound composition data (26 analytes). All panels show the same ordination (i.e. scatter of points) highlighting different sets of co-fermentations; here: Kveik strains. Notice the overlap among co-fermentations containing different Kveik strains (this Figure) is larger then the overlap among co-fermentations containing different NCY strains (Figure S1).

Non-highlighted co-fermentations are shown as grey circles; non-highlight confidence ellipses with hashed lines; size of symbols and characters is proportionate to total volatile aroma content of a co-fermentation. Percentages of total variation (RDA axes labels) is the proportion of total variance observed in the transformed VAC data which correlated with independent variables NCY strain and Kveik strain in RDA. NCY strains: B079 – *Hanseniaspora uvarum* B079 (blue); B143 – *Pichia kudriavzevii* B143 (magenta), B144 – *Meyerozyma guilliermondii* B144 (orange).

additional references (supplement data analysis)

Legendre, P.; Legendre. L. Numerical Ecology. Elsevier, Amsterdam, 2012, 3rd edition.

Legendre, P.; Gallagher, E.D. Ecologically meaningful transformations for ordination of species data. *Oecologica* **2001**, 29, 217-280.

Oksanen, J., Blanchet, F.G., Friendly, M., Kindt, R., Legendre, P., McGlinn, D., Minchin, P.R., O'Hara, R.B., Simpson, G.L., Solymos, P. and Stevens, M.H.H., **2020**. Vegan: Community ecology package. Ordination methods, diversity analysis and other functions for community and vegetation ecologists. R package version 2.5 (2019). R Package Version. Available online: https://cran.r-project.org/web/packages/vegan/index.html.
